# A Novel Invadopodia-Specific Marker for Invasive and Pro-Metastatic Cancer Stem Cells

**DOI:** 10.3389/fonc.2021.638311

**Published:** 2021-05-31

**Authors:** Shenq-Shyang Huang, Wen-Ying Liao, Chung-Chi Hsu, Tze-Sian Chan, Tai-Yan Liao, Pei-Ming Yang, Li-Tzong Chen, Shian-Ying Sung, Kelvin K. Tsai

**Affiliations:** ^1^ Graduate Program of Biotechnology in Medicine, Institute of Molecular and Cellular Biology, National Tsing Hua University, Hsinchu, Taiwan; ^2^ Laboratory of Advanced Molecular Therapeutics, Graduate Institute of Clinical Medicine, College of Medicine, Taipei Medical University, Taipei, Taiwan; ^3^ School of Medicine, College of Medicine, I-Shou University, Kaohsiung, Taiwan; ^4^ Division of Gastroenterology, Department of Internal Medicine, Wan Fang Hospital, Taipei Medical University, Taipei, Taiwan; ^5^ Integrated Therapy Center for Gastroenterological Cancers, Wan Fang Hospital, Taipei Medical University, Taipei, Taiwan; ^6^ Graduate Institute of Cancer Biology and Drug Discovery, College of Medical Science and Technology, Taipei Medical University, Taipei, Taiwan; ^7^ National Institute of Cancer Research, National Health Research Institutes, Tainan, Taiwan; ^8^ Department of Internal Medicine, Kaohsiung Medical University Hospital, Kaohsiung Medical University, Kaohsiung, Taiwan; ^9^ The Ph.D. Program for Translational Medicine, College of Medical Science and Technology, Taipei Medical University, Taipei, Taiwan; ^10^ Clinical Research Center, Wan Fang Hospital, Taipei Medical University, Taipei, Taiwan; ^11^ Taipei Medical University (TMU) and Affiliated Hospitals Pancreatic Cancer Groups, Taipei Medical University, Taipei, Taiwan

**Keywords:** ENO1, metastasis, cancer stem cells, invadopodia, prostate cancer, gastric cancer

## Abstract

**Introduction:**

Stem-like cancer cells or cancer stem cells (CSCs) may comprise a phenotypically and functionally heterogeneous subset of cells, whereas the molecular markers reflecting this CSC hierarchy remain elusive. The glycolytic enzyme alpha-enolase (ENO1) present on the surface of malignant tumor cells has been identified as a metastasis-promoting factor through its function of activating plasminogen. The expression pattern of surface ENO1 (sENO1) concerning cell-to-cell or CSC heterogeneity and its functional roles await further investigation.

**Methods:**

The cell-to-cell expression heterogeneity of sENO1 was profiled in malignant cells from different types of cancers using flow cytometry. The subcellular localization of sENO1 and its functional roles in the invadopodia formation and cancer cell invasiveness were investigated using a series of imaging, molecular, and *in vitro* and *in vivo* functional studies.

**Results:**

We showed here that ENO1 is specifically localized to the invadopodial surface of a significant subset (11.1%-63.9%) of CSCs in human gastric and prostate adenocarcinomas. sENO1^+^ CSCs have stronger mesenchymal properties than their sENO1^-^ counterparts. The subsequent functional studies confirmed the remarkable pro-invasive and pro-metastatic capacities of sENO1^+^ CSCs. Mechanistically, inhibiting the surface localization of ENO1 by downregulating caveolin-1 expression compromised invadopodia biogenesis, proteolysis, and CSC invasiveness.

**Conclusions:**

Our study identified the specific expression of ENO1 on the invadopodial surface of a subset of highly invasive and pro-metastatic CSCs. sENO1 may provide a diagnostically and/or therapeutically exploitable target to improve the outcome of patients with aggressive and metastatic cancers.

## Introduction

Metastasis is the major cause of cancer mortality. Despite advances in the understanding of the cellular and molecular pathways mediating cancer metastasis, the successful development of anti-metastasis therapies remains a highly unmet clinical need. For instance, human prostate adenocarcinoma (PAC) has a predilection to metastasize to the bone with more than 80% of patients who died from PAC developing bone metastases (1). Hormone therapy or chemotherapy has little effect on prolonging the survival of patients with metastatic PAC, resulting in a median overall survival of 1-2 years (2, 3). In advanced gastric adenocarcinoma (GAC), the majority of patients continue to have disease progression following treatments with chemotherapy and/or the anti-VEGF receptor 2 antibody (4). As such, a deeper mechanistic understanding of tumor aggressiveness and metastasis is crucial for further improving the outcome of patients with highly invasive and pro-metastatic cancer.

Mounting data over recent years, including genomic and single-cell sequencing analyses, have consistently indicated the existence of a subset of cancer cells termed cancer stem cells (CSCs), which are stem-like and serve as the driving force of cancer growth, metastasis, and treatment resistance (5–8). Interestingly, recent high-density genomic and lineage tracing studies further highlighted the tremendous heterogeneity and the plasticity of CSCs within the same tumor (5–7). The potential existence of a highly pro-metastatic subset of CSCs may be especially clinically important given that distant metastasis is the leading cause of patient mortality in advanced cancers (8–12). Whilst various populations of metastatic CSCs have been reported, including the CD44^+^CD24^-^ CSCs in breast cancer (13, 14), C-X-C motif chemokine receptor 4 (CXCR4)^+^ CSCs in pancreatic ductal adenocarcinoma (PDAC) (12), and CD26^+^ CSCs in colorectal cancer (11), most of these surface markers are not mechanism-informed and their molecular roles in the pro-metastatic capabilities of CSCs remain poorly understood. Equally unclear is whether there are ubiquitous or “tumor-agnostic” markers that mark pro-metastatic CSCs and can serve as diagnostic and/or therapeutic targets.

Invadopodia are the transformed version of podosomes expressed by motile cells such as macrophages, lymphocytes, dendritic cells, osteoclasts, endothelial cells, and smooth muscle cells (15–18). Invadopodia mediate focal degradation of extracellular matrix (ECM) by the localized proteolytic activity of proteases, especially matrix metalloproteinases (MMPs) (19, 20). Cancer cells use invadopodia during mesenchymal-type migration to degrade and invade ECM structures. Concordantly, a growing body of evidence reveals that invadopodia exist *in vivo* and may play a critical role in tumor invasion and metastasis (21–23). Invadopodia contribute to cancer cell invasion into the surrounding stroma, intravasation into the vasculature, and extravasation (20, 22, 24). Intravital imaging revealed invadopodia-like protrusions in tumor cells growing in the mammary fat pad of mice and those extending into the blood vessel wall or residing in perivascular niches (22, 23, 25). Complementing these observations, suppressing invadopodia by inhibiting Src, twist family BHLH transcription factor 1 (TWIST1), platelet-derived growth factor receptor alpha (PDGFR-α), TKS5 or a specific variant of ENAH actin regulator (MENA; MENA^INV^), has been shown to inhibit tumor metastasis in various tumor models (26). Moreover, caveolin 1 (CAV1), a constituent protein of caveolae, accumulates at invadopodia and its down-regulation inhibits invadopodia-mediated ECM degradation (27). Interestingly, CD44, a commonly used CSC marker, has been shown to contribute to invadopodia activity by promoting cortactin phosphorylation and recruits MMP14, implicating the potential link between invadopodia activity and CSCs (24, 28). The molecular basis underlying the relationship between cancer stemness and invadopodia activity and its significance in cancer metastasis awaits further investigations.

Many cellular enzymatic catalysts have evolved non-enzymatic functions. For instance, the expression of α-enolase (*ENO1*), a glycolytic enzyme that converts 2-phosphoglycerate into phosphoenolpyruvate, is upregulated in hypoxic conditions and cancer cells and is associated with poor prognosis in various types of cancers (29, 30). The alternative transcription of *ENO1* produces a nuclear protein termed MBP1-like p37, which binds to the c-myc promoter and functions as a transcriptional repressor (31). Interestingly, a fraction (6-8%) of the ENO1 protein is present on the plasma membrane in lymphocytes, monocytic cells, and endothelial cells where it serves as a major plasminogen receptor (32–34). The binding of ENO1 leads to activation of plasminogen to plasmin by the proteolytic action of either tissue-type or urokinase-type plasminogen activators (34). Multiple lines of evidence reveal that ENO1 is also present on the plasma membrane of cancer cells in non-small cell lung, breast, and pancreatic cancers (35–37), where it activates the plasminogen activator receptor (uPAR)/plasminogen/MMP axis, resulting in collagen degradation and cell invasion (38). Echoing the role of surface ENO1 (sENO1) in cancer cell invasiveness, vaccination of the mice with ENO1 has been reported to elicit anti-tumor immune responses, thereby delaying tumor progression and extending survival (39). Consistently, function-inhibitory anti-ENO1 antibodies have been shown to inhibit tumor metastasis in animal models of lung and pancreatic cancers (38, 40).

By using a series of cellular subset and biochemical analyses, we uncovered the specific localization of sENO1 on the invadopodial surface of a small subset of highly invasive and pro-metastatic CSCs across a wide variety of human solid and liquid cancers. Mechanistic studies revealed that sENO1 contributes to the invadopodial formation and activity in CSCs and is indispensable for cancer metastasis. Our studies thus identified the first tumor-agnostic and mechanism-informed marker for the pro-metastatic subset of CSCs, which illuminates an additional level of hierarchy in cancer stemness and provides a diagnostically and/or therapeutically exploitable target to improve the outcome of patients with aggressive and metastatic cancers.

## Materials and Methods

### Cell Culture

Primary PAC-derived 22Rv-1 cells, metastatic PAC PC-3 cells, primary GAC-derived AGS cells, and metastatic GAC NCI-N87 and MKN-45 cells (American Type Culture Collection, Manassas, VA) were maintained in DMEM or RPMI1640 (Invitrogen, Carlsbad, CA) supplemented with 10% fetal bovine serum and antibiotics. All frozen stocks received were immediately expanded and aliquots were prepared and stored in liquid nitrogen for future use, and cells were maintained for no longer than 3 months. Cell line authentication was performed by Bioresource Collection and Research Center (Hsinchu, Taiwan).

### Confocal Imaging and Invadopodia Assay

Invadopodia formation was induced by plating cells onto the gelatin matrix (G1392, Sigma-Aldrich, St. Louis, MO) as described previously (26). The cells were seeded on gelatin for 3 hours or longer and then immunostained with anti-cortactin (4F11; Abcam, Cambridge, UK), and Alexa Fluor 647 phalloidin (staining for F-actin; Invitrogen) and evaluated the staining patterns using confocal imaging analysis using a Leica TCS SP5 confocal microscope system (Leica Microsystems GmbH, Wetzlar, Germany). The cortactin^+^F-actin^+^ puncta seen under a confocal microscope represent the cross-sections of invadopodia that protrude downwardly from the cell bodies. In selected experiments, to visualize the three-dimensional (3D) architecture of invadopodia, confocal images collected were transferred to Imaris™ version 9.5.0 (Bitplane, Belfast, UK). The surface function was used to create the 3D structure features of fluorescent staining and the surface grain size was set to 0.100μm to maximize the particle feature. The 3D crop function was used to retrieve the Y-Z section to obtain the invadopodia structure vertically. In other experiments, to profile the expression pattern of sENO1 on invadopodia, the cells seeded on gelatin were immunostained for sENO1 with rabbit polyclonal anti-ENO1 (OriGene, Rockville, MD), after which the cells were fixed with 4% formaldehyde and then immunostained for cortactin and F-actin as described above. The degradation of the gelatin matrix was evaluated by seeding the cells on fluorescein-conjugated gelatin (Invitrogen) or by immunostaining the matrix with anti-Col1-3/4C (collagen type I cleavage site; ImmunoGlobe Antikörpertechnik GmbH, Himmelstadt, Germany). The fluorescence intensity of the degraded gelatin or Col1-3/4C was quantified by Image J and calculated according to the equation: CTCF (corrected total cell fluorescence) = integrated density - (area of the selected cell × mean fluorescence of background readings). The anti-ENO1 polyclonal antibody (pAb; α-ENO1) was described before and purified from immunized New Zealand semi-lop white rabbits (38, 40).

### Dual-Chamber Invasion Assay

Cells were seeded on Transwell inserts (BD Biosciences, San Jose, CA) with a thin layer of collagen type I (BD Biosciences) in the presence of 10% FBS and allowed to invade across the collagen for 12 hours. The cells that invaded through the insert membrane were fixed, stained with SYTOX Green (Invitrogen), and counted using a fluorescence microscope.

### Isolation of Invadopodial Proteins

To isolate invadopodia proteins, cells were plated on the gelatin matrix for 3 hours to induce the formation of invadopodia. Cell bodies were sheared from the surface of the plates to leave the invadopodia embedded in the gelatin (41). The invadopodia protein and the cell body protein fractions were then solubilized in immunoprecipitation buffer.

### Gene Expression Manipulations

The sustained KD of *ENO1*, *CAV1*, or *HSP70* expression in cells was achieved by lentivirus-mediated RNA interference using validated shRNA oligonucleotides in the lentivector pLKO.1-puro (MISSION shRNA lentiviruses; Sigma-Aldrich, St. Louis, MO) according to the manufacturer’s protocol. The clones selected were: *ENO1* (TRCN0000029324 and TRCN0000029326), *CAV1* (TRCN0000011218 and TRCN0000008002), *HSP70* (TRCN0000011467 and TRCN0000008758) and non-target control (SHC002V). Multiple (4–6) rounds of lentiviral infections were carried out to achieve a satisfactory (>80%) KD effect as verified by qRT-PCR and IB analysis. Lentivirus was produced in Lenti-X 293T™ cells (Clontech/Takara Bio, Mountain View, CA, USA) using the packaging vectors pMD2.G (Addgene #12259) and psPAX2 (Addgene #12260) to boost viral titer.

### qRT-PCR and Immunoblotting (IB) Analysis

qRT-PCR analysis was performed on the amplified RNA using the LightCycler FastStart DNA MASTERPLUS SYBR Green I Kit and the LightCycler System (Roche Diagnostics GmbH, Rotkreuz, Switzerland) and the Applied Biosystems ViiA7 Real-Time PCR System (ThermoFisher Scientific, Waltham, MA) according to the manufacturer’s instructions. Oligonucleotide primers were designed using Primer Bank (http://pga.mgh.harvard.edu/primerbank/index.html). IB protein analysis was performed according to standard protocols. Antibodies used for IB experiments include anti-caveolin-1 (CAV1; E249, Abcam), anti-cortactin (4F11, Abcam), anti-heat shock protein 70 (HSP70; EPR16893, Abcam), anti-β-tubulin, and anti-β-actin (GeneTex, Irvine, CA). Proteins were revealed after SDS/PAGE and immunoblotting with the indicated antibodies.

### Flow Cytometry and Cell Sorting

Cells were dissociated, antibody-labeled (1-2 µg per 10^6^ cells × 1 hour), and resuspended in HBSS/2%FBS as previously described (42, 43). The antibodies used include rabbit polyclonal anti-ENO1 (OriGene) in conjunction with Alexa Fluor 488-anti-rabbit IgG (Invitrogen), APC-anti-CD44, PE-anti-CD90 (all from BD Biosciences), and/or PE-anti-CD133 (Biolegend, San Diego, CA). Flow cytometry was done used a FACSCalibur™ Flow Cytometer (BD Biosciences) and Attune NxT Flow Cytometer (ThermoFisher Scientific) with the electronic gating set according to cells stained with the corresponding isotype-matched control IgG. Cell sorting was performed using Influx Cell Sorter™ (BD Biosciences).

### Tumorsphere and Limiting Dilution Assays (LDA)

The tumorsphere assay for PAC cells was performed as previously described (44, 45). For LDA, cells were plated in limiting dilution (1000, 100, 10, and 2 cells per well) in 96-well plates in the respective culture media. The presence of spheres was evaluated after 5 days.

### Distant Metastasis Tumor Models

Highly metastatic GAC NCI-N87 or MKN-45 cells were lentivirally transduced a green fluorescence protein (GFP) and firefly luciferase (FF-Luc) fusion vector (UBC-EGFP-T2A-Luc; System Biosciences, Palo Alto, CA) and GFP-positive cells were enriched by FACS. GFP-positive cells were freshly sorted according to the expression of sENO1 and CD90, and a relatively small number (1 × 10^4^ cells) of the sENO1^+^ and sENO^-^ subset of CD90^+^ CSCs were injected into the splenic pulp of immunodeficient NOD/SCID mice (BioLASCO Taiwan, Taiwan) over 1 minute, followed by splenectomy and splenic vein ligation. The development of hepatic and/or peritoneal metastatic tumors was monitored by bioluminescence imaging (BLI) according to the manufacturer’s recommendations (IVIS Imaging System, Caliper Life Sciences, Hopkinton, MA). Protocols for animal care and experimentation were approved by the Institutional Animal Care and Use Committee of National Health Research Institutes (NHRIs), Taiwan, and were adhered to the NIH Guide for the Care and Use of Laboratory Animals.

### Bioinformatics Analysis

The relapse-free and the overall survival data of 359 GAC patients stratified based on the *ENO1* (Affymetrix ID: 201231_s_at) transcript level was downloaded from KM Plotter (http://kmplot.com/analysis/index.php?p=service&cancer=gastric). The survival data and the *ENO1* transcript level from the 140 PAC patients in the Taylor et al. dataset and the 281 PAC patients in the Sboner data set were downloaded from NCBI’s Gene Expression Omnibus (accession number GSE21032 and GSE16560).

### Statistical Analysis

The statistical programming language R (cran.r-project.org) and SPSS 10.0 software (SPSS, Chicago, IL) were used to conduct the statistical analysis of our data. A two-tailed Student’s t-test was used for simple significance testing. Survival curves were generated using the Kaplan-Meier method. The curves were plotted and compared using the log-rank test using the GraphPad Prism 5.02 software. The data from the LDA were analyzed and plotted using the ELDA software (http://bioinf.wehi.edu.au/software/elda/index.html). The likelihood ratio test and Chi-square test were used to assess the significance.

## Results

### sENO1 Marks a Subset of CSCs Across Different Types of Cancers

**Figure 1:**
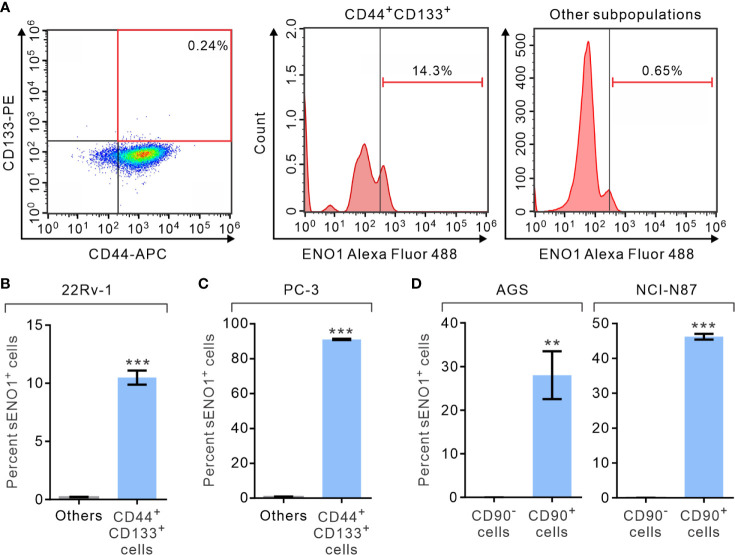
The expression of ENO1 on the cell-surface of a subpopulation of CSCs. **(A)** Representative FACS plots showing patterns of CD44, CD133, and surface ENO1 (sENO1) staining of primary prostate adenocarcinoma (PAC)-derived 22Rv-1 cells with the frequency of the boxed CD44^+^CD133^+^ cell population (representing CSCs in PAC; left) or sENO1^+^ cells in CD44^+^CD133^+^ CSCs (middle) or cells in the other subpopulations (representing non-CSCs; right) shown. **(B)** The percentages of sENO1^+^ cell subpopulation in CD44^+^CD133^+^ 22Rv-1 cells or cells in the other subpopulations (others). **(C)** The percentages of sENO1^+^ cell subpopulation in CD44^+^CD133^+^ PC-3 cells or cells in the other subpopulations. **(D)** The percentages of sENO1^+^ cell subpopulation in CD90^+^ gastric adenocarcinoma (GAC) AGS or NCI-N87 cells (representing CSCs in GAC) or CD90- cells (representing non-CSCs). Error bars represent mean ± SEM from three independent experiment (n = 3). Unpaired t-test was performed throughout where **p < 0.01; ***p < 0.001 in **(B–D)**.

We first analyzed the expression of ENO1 on the surface of PAC cells by fluorescence-assisted cell sorting (FACS) analysis. We measured the proportion of primary prostate cancer-derived 22Rv-1 cells that simultaneously expressed that surface markers CD44 and CD133, which have been shown to contain the enriched CSCs in PAC (46, 47). Remarkably, we uncovered that 22Rv-1 cells expressing sENO1 predominantly reside in the CD44^+^CD133^+^ subset of CSCs (**Figure 1A** and **Supplementary Figure 1**). Specifically, a significant proportion (10.5% on average) of CD44^+^CD133^+^ 22Rv-1 cells are positive for ENO1 while cells in the other subpopulations rarely (0.21% on average) express ENO1 on their cell surface (**Figure 1B**). Similarly, in metastatic PAC-derived line PC-3 cells, the majority (91.0% on average) of the CD44^+^CD133^+^ cells are positive for sENO1, whereas very few (0.9%) of the other subpopulations of cells express ENO1 on their cell surface (**Figure 1C**). To extend these findings to other types of cancers, we repeated the FACS analysis in primary tumor-derived AGS cells or liver metastatic GAC NCI-N87 cells, which affirmed that cells expressing sENO1 reside almost exclusively in the CD90^+^ cellular subset, which has been shown to contain the enriched CSCs in GAC (**Figure 1D**) (48). Likewise, sENO1^+^ cells were found predominantly in CD90^+^ cells in another metastatic GAC line MKN-45 cells, and in CD44^+^ GAC cells, which also contain the enriched CSCs in GAC (**Supplementary Figure 2**) (49). Taken together, these data suggest that sENO1 may mark a novel subset of CSCs in primary and metastatic cancer cells.

### sENO1^+^ CSCs Are Highly Invasive and Pro-Metastatic

**Figure 2:**
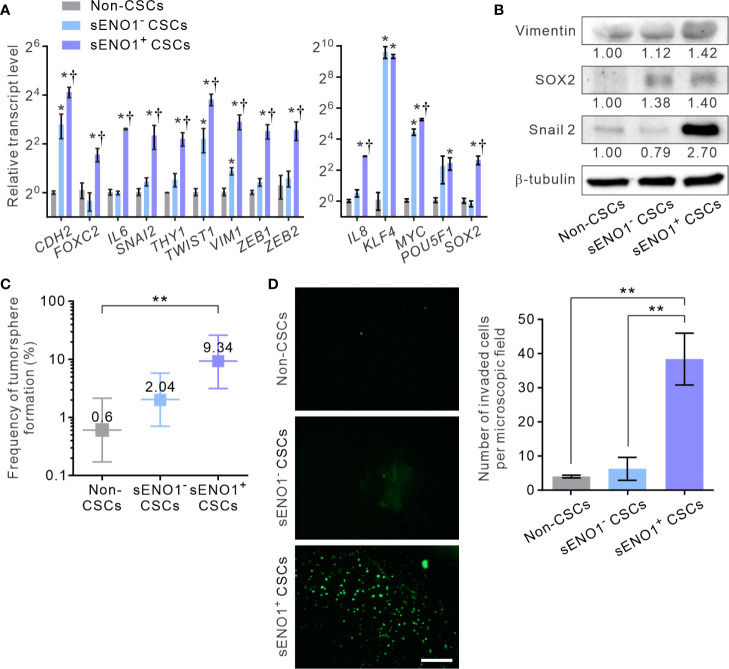
sENO1 marks a subpopulation of mesenchymal-like and highly invasive CSCs. **(A)** The relative transcript levels of the mesenchymal- (left) or pluripotency- (right) associated genes in sENO1^+^ CSCs (represented by CD44^+^CD133^+^ 22Rv-1 cells), sENO1^-^ CSCs, and non-CSCs (represented by cells in the other subpopulations) using qRT-PCR analysis. Error bars represent mean ± SEM from three independent experiments (n = 3). Unpaired t-test was performed throughout where *p < 0.05 versus non-CSCs; †p < 0.05 versus sENO1^-^ CSCs. **(B)** Immunoblotting analysis of the indicated markers selected from **(A)** in non-CSCs, sENO^+^, and sENO^-^ CSCs. Protein levels were quantified by densitometric analysis of the bands, normalized to β-tubulin (loading control). **(C)** Limiting dilution assay (LDA) demonstrating the tumorsphere-forming efficacy of each subset of tumor cells. Three independent experiments were performed (n = 6). Shown are maximum likelihood estimates with a 95% confidence interval, where **p < 0.01. **(D)** The invasive capacities of freshly sorted sENO1^+^ CSCs (represented by CD44^+^CD133^+^ 22Rv-1 cells), sENO1^-^ CSCs, and non-CSCs in 22Rv-1 cells in a dual-chamber invasion assay. Shown are representative immunofluorescence images of the invaded cells, with cell nuclei stained with SYTOX-green (green). Scale bars = 500 µm. Right, the number of invaded cells. Error bars represent mean ± SEM from three independent experiments (n = 3). Unpaired t-test was performed throughout where **p < 0.01.

Given that sENO1 is known to promote cell invasion through activating the uPA/uPAR/plasminogen axis (35–37), we posited that sENO1^+^ cells may comprise the pro-invasive subset of cancer cells. To address this possibility, we sorted the three subpopulations of cells, including CD44^+^CD133^+^sENO1^+^ cells (representing sENO1^+^ CSCs), CD44^+^CD133^+^sENO1^-^ cells (representing sENO1^-^ CSCs), and cells in other subpopulations from PAC 22Rv-1 cells and analyzed their expression of a panel of mesenchymal- and stemness-related genes using quantitative reverse transcription polymerase chain reaction (qRT-PCR) analysis. We uncovered that sENO1^+^ CSCs expressed extremely high (up to 1000-fold) transcript levels of EMT-associated genes, including *CDH2*, *FOXC2*, *IL6*, *SNAI2*, *THY1, TWIST1*, *VIM1*, *ZEB1*, and *ZEB2*, and stemness-associated genes, including *KLF4*, *MYC*, *POU5F1*, *SOX2*, and *IL8*, compared with those of non-CSCs (**Figure 2A**). In accordance with our speculation, sENO1^+^ CSCs expressed significantly higher (up to 4-fold) levels of these genes than those of sENO1^-^ CSCs. Concordantly, at the protein level, sENO^+^ CSCs expressed higher levels of the mesenchymal marker vimentin, the stemness factor SRY-Box Transcription Factor 2 (SOX2), and the epithelial-to-mesenchymal transition (EMT) regulator Snail 2, than those of sENO^-^ CSCs (**Figure 2B**). These data collectively indicate that sENO1^+^ CSCs are much more mesenchymal- and stem-cell-like than their sENO1^-^ counterpart.

To functionally verify the cellular and molecular data, we compared the tumorsphere-forming and the matrix-invasive capabilities of sENO1^+^ or ENO1^-^ CSCs. Indeed, whilst both sENO1^+^ and sENO1^-^ CSCs were capable of forming tumorspheres in low-attachment surface, a functional surrogate for the stemness and the tumorigenicity of tumor cells, sENO1^+^ cells were significantly more proficient in doing so (**Figure 2C**). Importantly, in keeping with the strong mesenchymal property of sENO1^+^ cells, they were considerably more proficient in invading through collagen than their sENO1^-^ counterparts. By contrast, the cells in other subpopulations could barely invade the collagen matrix (**Figure 2D**).

**Figure 3:**
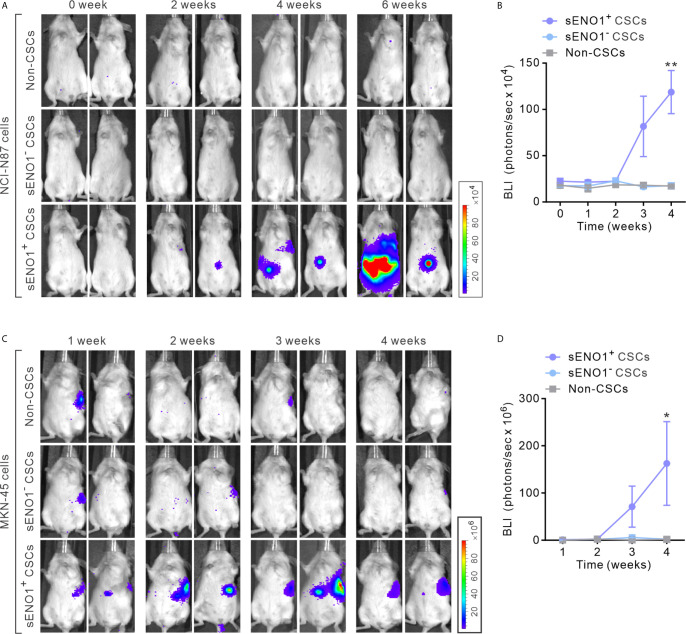
sENO1^+^ CSCs are highly pro-metastatic. **(A)** Representative BLI of NOD/SCID mice receiving an intra-splenic injection of sENO1^+^ CSCs (represented by CD90^+^ NCI-N87 cells), sENO1^-^ CSCs (CD90^-^ NCI-N87 cells), and non-CSCs (represented by CD90^-^ cells). at the indicated time following cell inoculation. **(B)** Tumor bulk quantified as BLI normalized photon counts as a function of time. Error bars represent mean ± SEM from one experiment (n = 8 mice per group). Unpaired t-test was performed throughout where **p < 0.01 versus non-CSCs. **(C)** Representative BLI of NOD/SCID mice receiving intra-splenic injection of sENO1+ CSCs (represented by CD90+ MKN-45 cells), sENO1- CSCs (CD90- MKN-45 cells) and non-CSCs (represented by CD90- cells). at the indicated time following cell inoculation. **(D)** Tumor bulk quantified as BLI normalized photon counts as a function of time. Error bars represent mean ± SEM from one experiment (n = 8 mice per group). Unpaired t-test was performed throughout where *p < 0.05 versus non-CSCs.

To explore the *in vivo* significance of the preceding findings, we stably transduced metastatic GAC NCI-N87 cells with firefly luciferase (FF-Luc), sorted sENO1^+^CD90^+^ (representing sENO1^+^ CSCs), sENO^-^CD90^+^ (representing sENO1^-^ CSCs) or CD90^-^ GAC NCI-N87 cells. We then injected a relatively small number (1 × 10^4^) of the cells into the splenic pulp of NOD/SCID mice and monitored the dissemination pattern of the tumor cells. As anticipated, sENO1^+^ CSCs developed extensive liver and peritoneal metastases, whereas sENO1^-^ CSCs or CD90^-^ non-CSCs failed to establish metastatic lesions (**Figures 3A, B**). Consistently, sENO1^+^ CSCs in another metastatic GAC line, MKN-45 cells, were highly pro-metastatic compared with their sENO1^-^ counterparts or non-CSCs (**Figures 3C, D**). This finding underscores the tremendous pro-metastatic capability of sENO1^+^ CSCs.

To explore the potential clinical relevance of the preceding experimental findings, we conducted clinical correlative analysis on the transcript level of *ENO1* with the prognosis in several large cohorts of patients with GAC or PAC. Whilst the total expression level of ENO1 may not directly reflect that in CSCs or the level of sENO1, the data showed a significantly (*P* < 0.0001) inverse correlation of the expression level of *ENO1* with both the relapse-free and the overall survival in patients with GAC (**Supplementary Figure 3A**) or PAC (**Supplementary Figure 3B**). Taken together, our data highlight the functional heterogeneity of different subsets of CSCs, and suggest that sENO1 marks a novel subset of highly tumorigenic and pro-metastatic CSCs.

### sENO1 Is Specifically Localized to the Invadopodial Surface of CSCs

**Figure 4:**
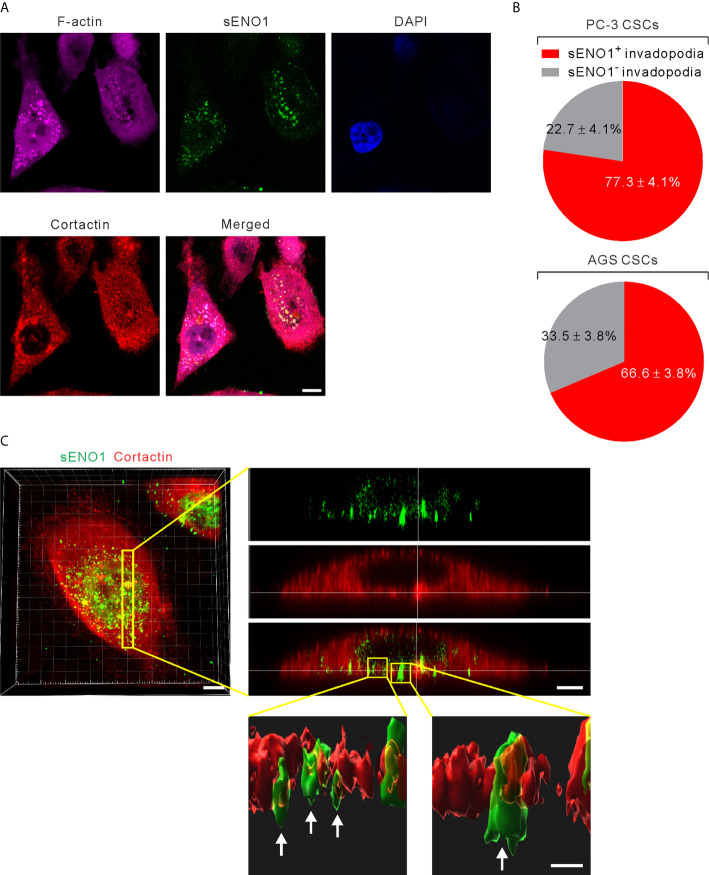
ENO1 is expressed on the invadopodial surface of CSCs. **(A)** Confocal views of PAC CSCs (represented by CD44^+^CD133^+^ PC-3 cells) showing the cross-section of invadopodia structures (represented by cortactin^+^F-acin^+^ puncta) with the colocalized surface ENO1 (sENO1; green), cortactin (red), and F-actin (magenta) that penetrate into the underlying gelatin matrix. Nuclei were counterstained with 4’,6-diamidino-2-phenylindole (DAPI; blue). Scale, 10 µm. **(B)** Top, a pie chart showing the percentage of sENO1^+^ invadopodia per PC-3 CSC. Bottom, a pie chart showing the percentage of sENO1^+^ invadopodia per GAC AGS CSC (represented by CD90^+^ AGS cells). **(C)** Left, representative three-dimensional (3D) reconstructed confocal image of CD44^+^CD133^+^ PC-3 CSCs showing the co-localization of sENO1 (green) and cortactin (red) at the ventral side of cell. Scale, 8 µm. Right upper, digital zoom-in image from serial Z sections (yellow rectangle) showing the spatial colocalization of sENO1 (green) and cortactin (red) at invadopodia. Scale, 5 µm. Right lower, the orthogonal view of the magnified areas (yellow squares at top) shown the distribution and localization of sENO1 and cortactin at the base of invadopodia. 3D rendered images of the invadopodia (arrows) were processed by using Imaris software. Scale, 1 µm.

It has been shown that ENO1 stably localizes to the lipid raft caveolae where it colocalizes with its constituent protein CAV1 (50). Since CAV1 accumulates in invadopodia whereby regulates the invadopodial biogenesis and ECM degradation (27, 51), we considered the possibility that sENO1 may also localize to the invadopodial surface of CSCs. We freshly sorted CD44^+^CD133^+^ CSCs from PC-3 cells and induced invadopodial formation by plating them on the gelatin matrix. We then immunostained the cells with ENO1 before the fixation and membrane permeabilization (to specifically detect sENO1) for the subsequent staining with the invadopodial markers cortactin and F-actin. We quantified the percentage of invadopodia that co-express sENO1, cortactin, and/or F-actin per cell, which revealed that the majority (77.3 ± 4.1%) of cortactin^+^F-actin^+^ invadopodia on CSCs also stained positively with sENO1 (**Figures 4A, B**). Concordantly, we examined the expression pattern of sENO1 in CD90^+^ CSCs in GAC AGS cells and found that indeed the majority (66.6%) of the invadopodia are positive for sENO1 expression in GAC cells (**Figure 4B**). Notwithstanding the prominent colocalization of sENO1 and the hallmark invadopodia marker cortactin in two-dimensional confocal images, Imaris 3D rendered analysis of the confocal images revealed that sENO1 is mainly localized to the tip and the body of the invadopodia of CSCs, whereas cortactin localization is restricted to the base of the invadopodia (**Figure 4C**).

**Figure 5:**
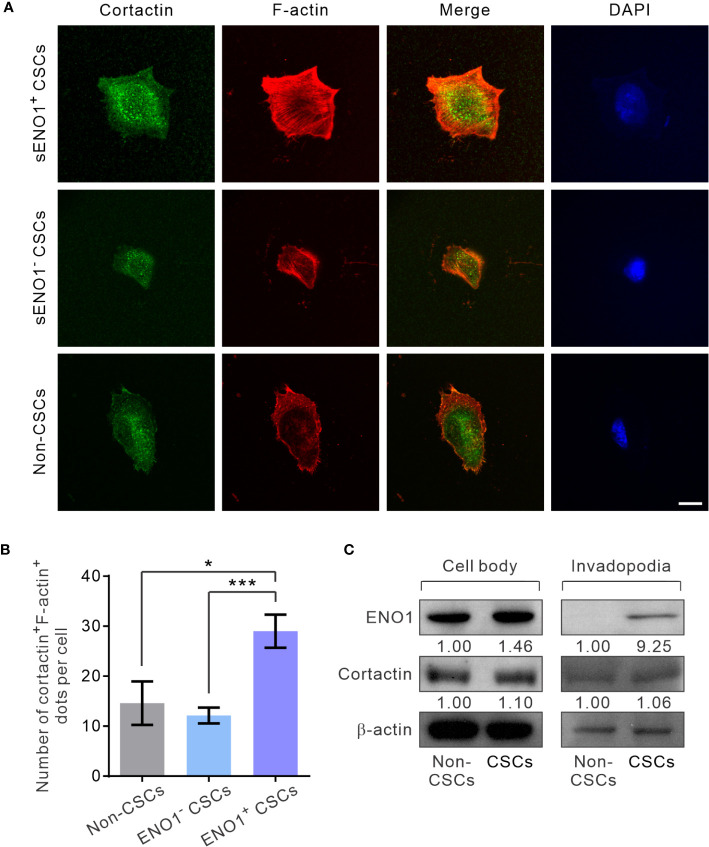
sENO1^+^ CSCs generate more invadopodia than their sENO1^-^ counterparts. **(A)** Confocal views of sENO1^+^ PC-3 CSCs (represented by CD44^+^CD133^+^ cells), sENO1^-^ CSCs, and non-CSCs (represented by cells in the other subpopulations) showing invadopodia (yellow puncta) with the colocalized cortactin (green) and F-actin (red) that penetrate the underlying gelatin matrix. Nuclei were counterstained with 4’,6-diamidino-2-phenylindole (DAPI; blue). Scale, 10 µm. **(B)** Quantification of the invadopodia density per cell in **(A)**. Error bars represent mean ± SEM from three independent experiments (n = 50 cells counted per sample). Unpaired t-test was performed where *p < 0.05, ***p < 0.001. **(C)** Representative immunoblots of ENO1, cortactin, and F-actin in the cell body (left) and the invadopodial (right) protein lysates fractionated from CD44^+^CD133^+^ PC-3 CSCs seeded on gelatin for 6 hours. Protein levels were quantified by densitometric analysis of the bands, normalized to β-actin (loading control).

Next, to substantiate the above findings, we sorted the three subpopulations of PAC PC-3 cells based on their expression pattern of sENO1 and the CSC markers CD44, and CD133 and induced the invadopodia formation. Remarkably, sENO1^+^ CSCs generated considerably more invadopodia than their sENO1^-^ counterparts or other cells (**Figures 5A, B**). To confirm these imaging analyses, we isolated the invadopodial protein from cells using a previously established fractionation protocol (41, 52). We fractionated PC-3 CSCs seeded on the gelatin matrix into the invadopodia fraction and the cell body fraction and probed the expression of ENO1 using immunoblotting analysis. Since the anti-ENO1 antibody does not distinguish between cytosolic ENO1 and sENO1, we found that the majority of the protein was expressed in the cell body and only a small amount of the protein was detected in the invadopodial lysate of CSCs. Notably and importantly, we could barely detect any ENO1 protein in the invadopodial lysate of non-CSCs (**Figure 5C**), affirming that sENO1 is a CSC-specific invadopodia marker.

### sENO1 Is Functional Important for the Invadopodia and the Invasiveness of CSCs

**Figure 6:**
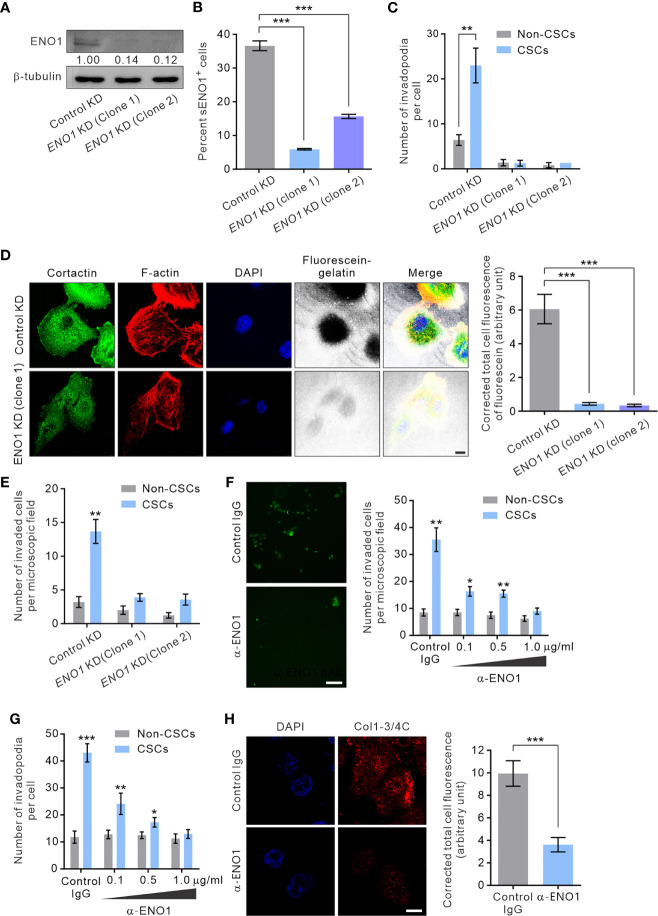
sENO1 contributes to the invadopodial formation and the matrix-degradative function of CSCs. **(A)** Immunoblotting analysis showing the effect of lentivirus shRNA-mediated knockdown (KD) of ENO1 expression in PC-3 cells. Protein levels were quantified by densitometric analysis of the bands, normalized to β-tubulin (loading control). **(B)** Bar graph showing the percentage of sENO1^+^ PC-3 cells with KD of ENO1 expression or control KD. **(C)** Bar graph showing the density of invadopodia (represented by cortactin^+^F-actin^+^ puncta) per cells in PC-3 CSCs (represented by CD44^+^CD133^+^ cells) or non-CSCs (represented by cells in other subpopulations) with ENO1 KD or control KD. Error bars represent mean ± SEM from three independent experiments (n = 3). Unpaired t-test was performed where **p < 0.01, ***p < 0.001 in **(B, C)**. **(D)** PC-3 cells with KD of ENO1 expression or the control KD cells were seeded on top of a fluorescein-conjugated gelatin matrix and immunostained with cortactin (green) or phalloidin (F-actin; red). Nuclei were counterstained with DAPI (blue). Right, the fluorescence intensity of fluorescein-conjugated gelatin within the boundary (determined by F-actin staining) of PC-3 cells with ENO1 KD or control KD (n = 50 cells counted per sample). Unpaired t-test was performed where ***p < 0.001. **(E)** Bar graph showing the invasive capacity of PC-3 CSCs with ENO1 KD or control KD in a dual-chamber invasion assay. Error bars represent mean ± SEM from three independent experiments (n = 3). Unpaired t-test was performed where **p < 0.01 versus non-CSCs. **(F)** Representative immunofluorescence images of CD44^+^CD133^+^ PC-3 cells (representing CSCs) that had invaded the type I collagen matrix in the presence of an increasing concentration (0.1-1.0 µg/ml) of the anti-ENO1 polyclonal antibody (pAb; α-ENO1) in a dual-chamber invasion assay. The nuclei of the invaded cells were stained with SYTOX-green. Scale bars, 500 µm. Right, the number of invaded cells. Cells in other subpopulations (representing non-CSCs) were included as a control. Error bars represent mean ± SEM from three independent experiments (n = 3). Unpaired t-test was performed throughout where *p < 0.05, **p < 0.01 versus non-CSCs. **(G)** The invadopodia density per cell in PC-3 CSCs or non-CSCs exposed to an increasing concentration of α-ENO1. Error bars represent mean ± SEM from three independent experiments (n = 50 cells counted per sample). Unpaired t-test was performed where *p < 0.05, **p < 0.01, ***p < 0.001 versus non-CSCs. **(H)** PC-3 CSCs were seeded on top of a gelatin matrix in the presence or absence of α-ENO1 (20 µg/ml). Shown are the extent of matrix degradation as reflected by immunostaining with anti-Col1-3/4C (red). Right, the total cell fluorescence intensity of Col1-3/4C in PC-3 CSCs treated with α-ENO1 or a control IgG (n = 50 cells counted per sample). Unpaired t-test was performed where ***p < 0.001.

To gain mechanistic insight into the potential role of sENO1 in the invadopodial functions in CSCs, we stably downregulated the expression of *ENO1* using lentivirus-mediated transduction of small hairpin RNA (shRNA) in PAC PC-3 cells. We were able to substantially reduce the expression of *ENO1* using two independent shRNA (**Figure 6A**). Of note, the ENO1-deficient cells were cultivated in the presence of sodium pyruvate (100 µM) to salvage their glycolytic activity, thereby maintaining their viability following knockdown (KD) of *ENO1* expression (**Supplementary Figure 4**). We confirmed that KD of *ENO1* expression substantially shrank the population of sENO1^+^ cells (**Figure 6B**). Importantly, KD of *ENO1* expression considerably and preferentially diminished the number of invadopodia present on CSCs (**Figure 6C**). Functional verification of the finding was provided by the substantial reduction in the degradation of the gelatin matrix surrounding and beneath CSCs when *ENO1* expression was knocked down (**Figure 6D**). Echoing the impaired invadopodia formation in ENO1-deficient CSCs, KD of *ENO1* expression preferentially reduced the invasive capacity of CSCs in PC-3 cells; by contrast, non-CSCs had much less invasive capacity than CSCs either with or without *ENO1* KD (**Figure 6E**). The functional importance of ENO1 in CSC-mediated metastasis gained further support from a distant metastasis model of GAC, wherein KD of *ENO1* expression significantly attenuated the pro-metastatic capability of CSCs (**Supplementary Figure 5A**).

Whilst KD of *ENO1* expression yielded results consistent with the functional importance of ENO1 in the invadopodia formation and the invasive capacity of CSCs, this approach did not distinguish between the role of sENO1 with that of cytosolic ENO1, which predominantly serves as a glycolytic enzyme. To clarify this, we raised a function-blocking polyclonal antibody (pAb) that has been shown to block the interaction of ENO1 with plasminogen and its pro-migratory ability for cancer cells (32, 53), enabling us to specifically determine the functional importance of sENO1 in the invadopodia and the invasiveness of CSCs. In keeping with the specific localization of sENO1 on the invadopodia of CSCs, using a dual-chamber invasion assay, we confirmed that the functional inhibition of sENO1 could selectively and dose-dependently inhibit the invasive behavior of CD44^+^CD133^+^ CSCs without significantly affecting that of non-CSCs (**Figure 6F**). As such, the anti-ENO1 pAb specifically attenuated the ability of CSCs to form invadopodia (**Figure 6G**) or to degrade the surrounding collagen matrix (**Figure 6H**). These data collectively point to the functional importance of sENO1 in the invadopodia and the invasiveness of CSCs.

### CAV1-Mediated Localization of sENO1 Is Indispensable for Invadopodia Formation and Cancer Cell Invasiveness

**Figure 7:**
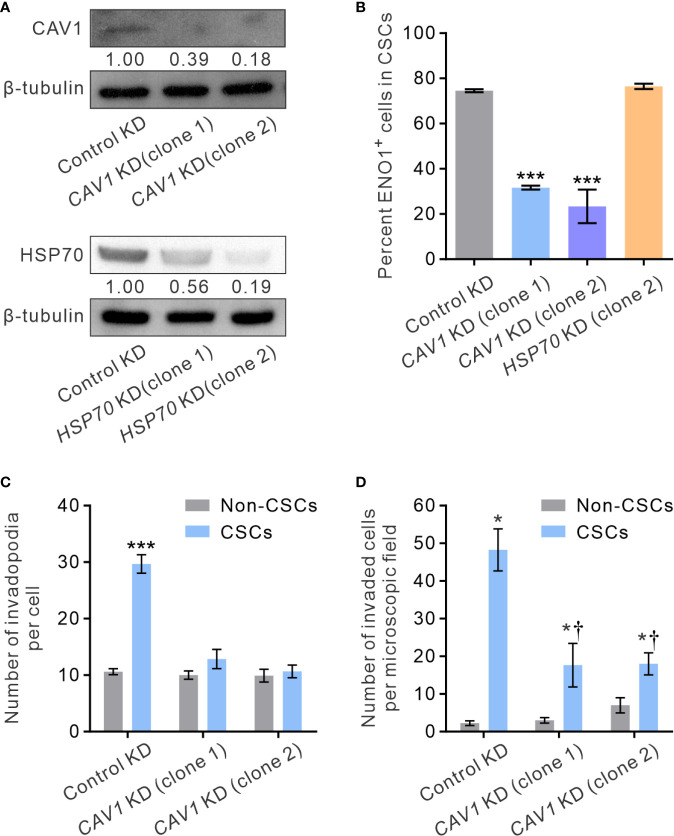
CAV1 is indispensable for the surface localization of sENO1 on CSCs and its pro-invadopodia and pro-invasive functions. **(A)** Immunoblotting analysis showing the effect of lentivirus shRNA-mediated knockdown (KD) of CAV1 (top) or HSP70 (bottom) expression in PC-3 cells. Protein levels were quantified by densitometric analysis of the bands, normalized to β-tubulin (loading control). **(B)** Bar graph showing the percentage of sENO1^+^ cells in PC-3 CSCs (represented by CD44^+^CD33^+^ cells) with KD of CAV1 or HSP70 expression or control-KD. Unpaired t-test was performed throughout where ***p < 0.001 versus control KD. **(C)** Bar graph showing the density of invadopodia (represented by coractin^+^F-actin^+^ puncta) per cell in PC-3 CSCs or non-CSCs (represented by cells in other subpopulations) with CAV1 KD or control KD. Unpaired t-test was performed throughout where ***p < 0.001. **(D)** Bar graph showing the invasive capacity of PC-3 CSCs or non-CSCs with CAV1 KD or control KD in a dual-chamber invasion assay. Error bars represent mean ± SEM from three independent experiments (n = 3). Unpaired t-test was performed throughout where *p < 0.05 versus non-CSCs; †p < 0.05 versus control KD.

It has been shown that ENO1 is transported to the cell surface in response to external stimuli, including epithermal growth factor or lipopolysaccharide exposure (54). The transmembranous transport of ENO1, which lacks a signal sequence that can direct it across the endoplasmic reticulum-Golgi export pathway, involves non-classical molecular processes, such as HSP70. Alternatively, in highly metastatic breast cancer MDA-MD-231 cells that express abundant sENO1, the expression of sENO1 is found dependent on its association with the caveolae proteins CAV1 and to a lesser extent annexin 2 (50). To understand the mechanisms by which sENO1 is localized to the surface of CSCs and to corroborate the functional role of sENO1 in invadopodia and CSC invasiveness, we stably downregulated the expression of *CAV1* or *HSP70* in PC3 cells and examined the expression level of sENO1 on CSCs (**Figure 7A**). Interestingly, KD of *CAV1* expression markedly reduced the expression level of sENO1 on CD44^+^CD133^+^ PC3 cells (representing CSCs), whereas KD of *HSP70* expression did not significantly affect the percentage of CSCs expressing sENO1 (**Figure 7B**). In keeping with the specific role of sENO1 in the invadopodia formation in CSCs, KD of *CAV1* expression substantially reduced the number of invadopodia on CSCs to a level similar to non-CSCs (**Figure 7C**). Notably, the density of invadopodia on non-CSCs was not significantly affected by CAV1 downregulation. Echoing the change in the invadopodia density, KD of *CAV1* expression specifically attenuated the invasive capacity of CSCs in a dual-chamber invasion assay, whereas the invasive capacity of non-CSCs was low and not affected by *CAV1* KD (**Figure 7D**). Consistently, KD of *CAV1* expression markedly attenuated the pro-metastatic capability of CSCs in a distant metastasis model of GAC to an extent similar to that achieved by downregulating *ENO1* expression (**Supplementary Figure 5B**). These data collectively suggest that CAV1-mediated cell-surface expression of ENO1 is critical to the invadopodia formation and the invasive and pro-metastatic capacities of CSCs.

## Discussions

The CSC model of tumorigenesis maintains that tumors are hierarchically organized and only a small population of cancer cells with the self-renewing ability of stem cells can initiate and sustain tumors (55, 56). As CSCs are capable of self-renew and sustaining tumorigenesis, they are also likely to be the major driving force of cancer dissemination and distant metastasis (55, 56). Interestingly, emerging data in breast cancer suggests that there could be a further level of hierarchy in CSCs concerning their ability to proliferate or metastasize to distant organs (13, 57). The earliest evidence of the CSC heterogeneity was provided by the identification of a population of CXCR4^+^ CSCs in PDAC that are highly migratory and are capable of initiating distant metastasis (12). Later, by comparing the CD44^+^CD24^-^ and the aldehyde dehydrogenase 1 (ALDH1)-positive populations of breast cancer cells, Liu et al. proposed that breast CSCs may exist in alternative mesenchymal-like and epithelial-like states which can transition between each other (13). Notably and importantly, the transition between the two CSC states is mediated epigenetically either by the tumor microenvironment through cytokine and chemokine signaling or by the differential expression of microRNAs (58, 59). Specifically, mesenchymal-like CSCs express EMT-associated genes and are mostly quiescent while epithelial-like CSCs express epithelial markers and are highly proliferative. In keeping with this notion, Stankic et al. reported that the inhibitor of DNA binding 1 (ID1)-dependent transcriptional repression of TWIST1 converts metastatic breast cancer cells from an EMT to a mesenchymal-epithelial transition state, and this phenotypic conversion is required for their metastatic colonization in the lung (57). Furthermore, it has been shown that the long-term progression of PDAC is mediated by distinct subsets of CSCs in temporally restricted bursts with little overlap between subsequent generations (60). The potential existence of a highly pro-metastatic subset of CSCs may be especially clinically important given that distant metastasis is the leading cause of patient mortality in advanced cancers (8, 9, 11, 12). Thus, identifying markers of invasive and pro-metastatic subsets of CSCs and their molecular targeting may provide a new avenue for treating and/or preventing cancer metastasis.

Notwithstanding the various cell-surface markers currently used to enrich for CSCs from different types of malignant tumors, only very few of them inform the mechanisms underpinning their tumorigenic and/or pro-metastatic potentials. The identification of sENO1 as a marker of highly invasive and pro-metastatic CSCs in multiple types of malignant tumors not only emphasizes the functional heterogeneity within CSCs but also supports the existence of pro-metastatic CSCs. Importantly, the functional role of sENO1 in the formation and the proteolytic function of invadopodia on CSCs provides the hitherto first molecularly tractable mechanism that links CSCs, invadopodia, and cancer metastasis.

Whilst ENO1 is originally identified as a critical glycolytic enzyme whose expression is upregulated in malignant tumors (29), like many metabolic enzymes, ENO1 evolves other cellular functions independent of its glycolytic function. For instance, a nuclear protein MBP1-like p37, produced by the transcriptional variant of *ENO1*, functions as a transcriptional repressor of the *MYC* gene (31). Most notably, a significant proportion of the ENO1 protein redistributes to the surface of cancer cells, wherein it evolves proteolytic functions through activating the uPAR/plasminogen/MMP axis (38), thereby contributing to tumor progression in particular the process of metastasis. It has been shown that metastatic cancer cells are enriched for those expressing sENO1 in an orthotopic mouse PDAC model (40). Furthermore, sENO1 has been shown to elicit a specific T-cell response in patients with PDAC, and sENO1-specific T cells could inhibit the growth of xenotransplanted human pancreatic tumors (35). Consistently, in the Kras^G12D^/Trp53^R172H^/Cre mouse model of PDAC, vaccination of the mice with the DNA of ENO1 elicits significant anti-tumor immune responses, thereby delaying tumor progression and extending survival (39). The functional importance of sENO1 in tumor progression was further validated by the ability of a function-inhibiting anti-ENO1 antibody, which suppresses cell-associated plasminogen activation and matrix metalloproteinase activation and thereby inhibits matrix degradation and cell invasion, to inhibit tumor metastasis in various animal models of lung cancer and PDAC (38, 40). In another therapeutic approach, an ENO1-binding peptide, when conjugated with chemotherapeutic agents such as doxorubicin or vinorelbine, could exhibit an enhanced antitumor effect in a mouse model of human colorectal cancer (61). Our results echo these prior findings and additionally reveal the specific localization of sENO1 on the invadopodial surface of a subset of CSCs, wherein it critically contributes to their pro-invasive and pro-metastatic capabilities.

Intriguingly, ENO1 might not be the only metabolic enzyme that is found in invadopodia. Previous proteomic analysis has identified that many glycolytic enzymes, including such as glyceraldehyde-3-phosphate dehydrogenase (GAPDH), ENO1, muscle pyruvate kinase (PKM2), phosphoglycerate mutase 1 (PGK1), lactate dehydrogenase (LDH), isocitrate dehydrogenases 1 (IDH1), glucose 6 phosphate dehydrogenase (G6PD), and aldehyde dehydrogenase (ALDH), are enriched in the invadopodia lysate of human melanoma cells (52). Whilst cancer cells are known to constitutively upregulate the expression of glycolytic enzymes, including ENO1, as a part of the “Warburg effect”, it remains unclear why these enzymes are concentrated at invadopodia. One potential explanation is the potential interactions between glycolytic activity and the cytoskeletal organization (62). On the other hand, glycolytic enzymes, including enolase, have been frequently found in association with membranes (63), raising the possibility that glycolytic activity and pentose phosphate pathway may be involved in the redox control, membrane trafficking, and the cytoskeletal remodeling that are required for the invadopodial biogenesis. Our findings shed new light on the specific localization of ENO1 to invadopodia, which can be at least partially attributed to its novel function of inducing peri-invadopodial proteolysis rather than its role as a glycolytic enzyme. This raised the intriguing possibility that other invadopodia-localized metabolic enzymes may also develop the novel function of facilitating the biogenesis and/or the activity of invadopodia. Further in-depth mechanistic studies are warranted to address this possibility.

Our findings of the important role of CAV1 in the specific redistribution of ENO1 to the invadopodial surface of CSCs accord with the reported interaction between ENO1 and CAV1 and the role of CAV1 in ENO1-induced cell migration (50). CAV1 is a resident protein of caveolae, which is a special type of lipid rafts required for the assembly and function of invadopodia in cancer cells. As such, CAV1 has been reported to accumulate at invadopodia and plays a role in the related ECM degradation (27). Consistently, depletion of CAV1 disrupts the association of essential components of invadopodia/podosomes, including Src kinases, β1-integrin and uPAR, thereby compromising the migration of cells on ECM (51). Our finding that CAV1 is indispensable for the localization of sENO1 on the invadopodial surface reinforces the functional role of lipid rafts, specifically caveolae, in invadopodia. Our functional studies further underpin the roles of CAV1 expression in the invadopodia formation and the invasive capacity of CSCs and their pro-metastatic capability. Notwithstanding these consistent findings, given that CAV1 and caveolae are known to regulate diverse signaling pathways involved in cell adhesion and assorted cellular functions (51, 64), a compelling question raised from our study is whether sENO1 plays a specific and dominant role in CAV1-regulated cancer invasiveness and metastasis. Further in-depth mechanistic studies are warranted to delineate the epistatic relationship between CAV1, sENO1, and other signaling pathways. Why sENO1 is specifically localized on the invadopodial surface of CSCs and how CAV1 and caveolae regulate this CSC-specific process also await further investigations.

The expression level of ENO1 has been found upregulated in various cancers and is associated with poor prognosis, including non-small cell lung cancer and head and neck cancer (29, 30, 65). ENO1 expression has also been associated with tumor de-differentiation and venous invasion in hepatocellular carcinoma (66). Intriguingly, a study has reported that the presence of circulating auto-antibodies against ENO1 was associated with better clinical outcomes in patients with advanced pancreatic cancer (67). Our study extended these findings to other types of solid tumors, including GAC and PAC, wherein the transcript level of *ENO1* correlates with the poor prognosis of patients. Notwithstanding these clinical correlations, it should be noted that the transcript analysis from tumor samples neither reflects the expression pattern of ENO1 in the small subpopulation of CSCs nor the amount of sENO1 protein present on the invadopodial surface. Further elucidation of the prognostic role of sENO1 in human cancers may require the flow cytometric analysis of sENO1 on cells isolated from freshly resected human tumors or the use of diagnostic antibodies that specifically recognize sENO1 but not its intracellular counterparts, which awaits further development.

In conclusion, our study identified the specific expression of ENO1 on the invadopodial surface of a novel subset of highly invasive and pro-metastatic CSCs across different types of cancer. Our results lend support to the emerging paradigm in which a specific subset of CSCs preferentially promote tumor aggressiveness and metastasis. We envisage that sENO1 may provide a diagnostically and/or therapeutically exploitable target to improve the outcome of patients with aggressive and metastatic cancers.

## Data Availability Statement

The original contributions presented in the study are included in the article/**Supplementary Material**. Further inquiries can be directed to the corresponding author.

## Ethics Statement

The animal study was reviewed and approved by Institutional Animal Care and Use Committee of National Health Research Institutes, Taiwan.

## Author Contributions

S-SH, C-CH, and T-YL conducted molecular and biochemical experiments. W-YL and S-YS performed animal studies. T-SC and L-TC interpreted data, acquired funding and helped prepare the manuscript. P-MY performed bioinformatics analyses. KT supervised the research, acquired funding, and prepared the manuscript. All authors contributed to the article and approved the submitted version.

## Funding

This work was supported in part by the Ministry of Science and Technology, Taiwan (MOST 108-2314-B-038-105, MOST-109-2314-B-038-130, and MOST 109-2327-B-038-001 to KKT; MOST 104-TDU-M-212-00005 to LTC; MOST 107-2314-B-038-103 to T-SC).

## Conflict of Interest

The authors declare that the research was conducted in the absence of any commercial or financial relationships that could be construed as a potential conflict of interest.
