# Corrigendum: A Novel Invadopodia-Specific Marker for Invasive and Pro-Metastatic Cancer Stem Cells

**DOI:** 10.3389/fonc.2021.718849

**Published:** 2021-06-22

**Authors:** Shenq-Shyang Huang, Wen-Ying Liao, Chung-Chi Hsu, Tze-Sian Chan, Tai-Yan Liao, Pei-Ming Yang, Li-Tzong Chen, Shian-Ying Sung, Kelvin K. Tsai

**Affiliations:** ^1^ Graduate Program of Biotechnology in Medicine, Institute of Molecular and Cellular Biology, National Tsing Hua University, Hsinchu, Taiwan; ^2^ Laboratory of Advanced Molecular Therapeutics, Graduate Institute of Clinical Medicine, College of Medicine, Taipei Medical University, Taipei, Taiwan; ^3^ School of Medicine, College of Medicine, I-Shou University, Kaohsiung, Taiwan; ^4^ Division of Gastroenterology, Department of Internal Medicine, Wan Fang Hospital, Taipei Medical University, Taipei, Taiwan; ^5^ Integrated Therapy Center for Gastroenterological Cancers, Wan Fang Hospital, Taipei Medical University, Taipei, Taiwan; ^6^ Graduate Institute of Cancer Biology and Drug Discovery, College of Medical Science and Technology, Taipei Medical University, Taipei, Taiwan; ^7^ National Institute of Cancer Research, National Health Research Institutes, Tainan, Taiwan; ^8^ Department of Internal Medicine, Kaohsiung Medical University Hospital, Kaohsiung Medical University, Kaohsiung, Taiwan; ^9^ The Ph.D. Program for Translational Medicine, College of Medical Science and Technology, Taipei Medical University, Taipei, Taiwan; ^10^ Clinical Research Center, Wan Fang Hospital, Taipei Medical University, Taipei, Taiwan; ^11^ Taipei Medical University (TMU) and Affiliated Hospitals Pancreatic Cancer Groups, Taipei Medical University, Taipei, Taiwan

**Keywords:** ENO1, metastasis, cancer stem cells, invadopodia, prostate cancer, gastric cancer

In the original article, there was a mistake in the legend for **Figure 3** as published. The name of the mouse strain described in (A) and (C) was mis-spelled as “NOC/SCID”, which should be “NOD/SCID”. Appended below is the corrected legend.

**Figure 3:**
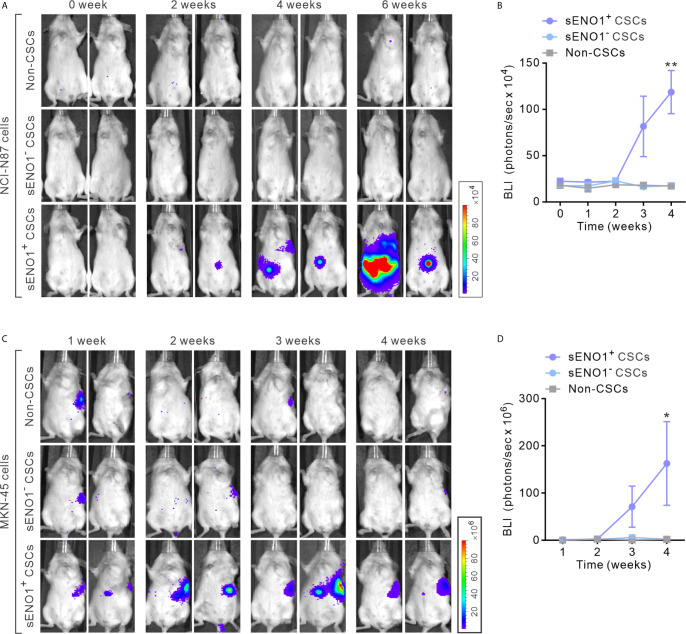
sENO1^+^ CSCs are highly pro-metastatic. **(A)** Representative BLI of NOD/SCID mice receiving an intra-splenic injection of sENO1^+^ CSCs (represented by CD90^+^ NCI-N87 cells), sENO1^-^ CSCs (CD90^-^ NCI-N87 cells), and non-CSCs (represented by CD90^-^ cells). at the indicated time following cell inoculation. **(B)** Tumor bulk quantified as BLI normalized photon counts as a function of time. Error bars represent mean ± SEM from one experiment (n = 8 mice per group). Unpaired t-test was performed throughout where **p < 0.01 *versus* non-CSCs. **(C)** Representative BLI of NOD/SCID mice receiving intra-splenic injection of sENO1^+^CSCs (represented by CD90^+^ MKN-45 cells), sENO1^-^ CSCs (CD90^-^ MKN-45 cells) and non-CSCs (represented by CD90^-^ cells). at the indicated time following cell inoculation. **(D)** Tumor bulk quantified as BLI normalized photon counts as a function of time. Error bars represent mean ± SEM from one experiment (n = 8 mice per group). Unpaired t-test was performed throughout where *p < 0.05 *versus* non-CSCs.

In the original article, there was a mistake in the legend for **Figure 4** as published. We mislabeled the name of the cell line in (B) as “NCI-N87”, which should be “AGS” as described in the main text. Appended below is the corrected legend.

**Figure 4:**
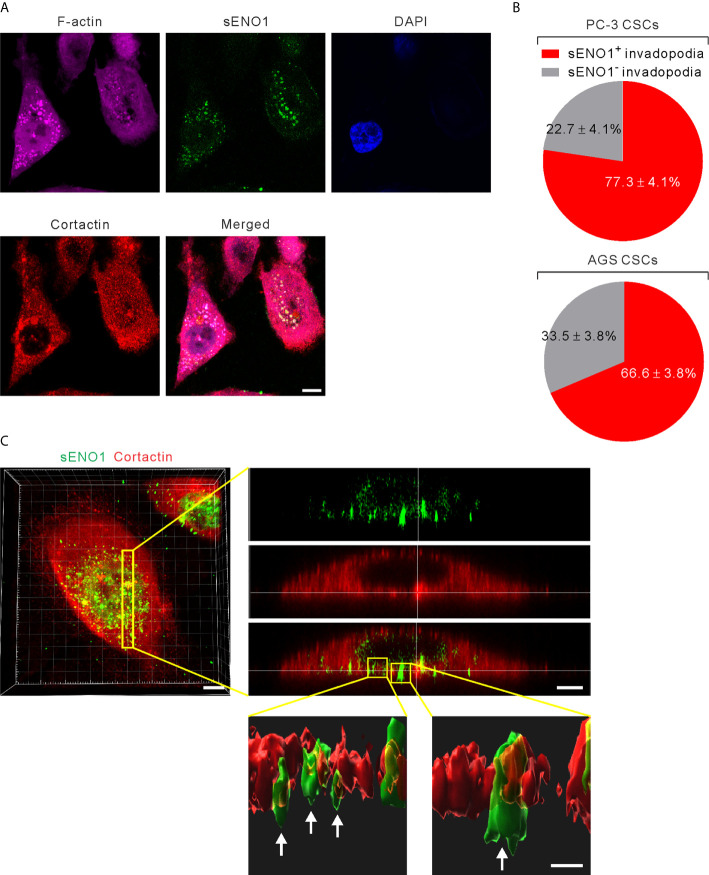
ENO1 is expressed on the invadopodial surface of CSCs. **(A)** Confocal views of PAC CSCs (represented by CD44^+^CD133^+^ PC-3 cells) showing the cross-section of invadopodia structures (represented by cortactin^+^F-acin^+^ puncta) with the colocalized surface ENO1 (sENO1; green), cortactin (red), and F-actin (magenta) that penetrate into the underlying gelatin matrix. Nuclei were counterstained with 4’,6-diamidino-2-phenylindole (DAPI; blue). Scale, 10 μm. **(B)** Top, a pie chart showing the percentage of sENO1^+^ invadopodia per PC-3 CSC. Bottom, a pie chart showing the percentage of sENO1^+^ invadopodia per GAC AGS CSC (represented by CD90^+^ AGS cells). **(C)** Left, representative three-dimensional (3D) reconstructed confocal image of CD44^+^CD133^+^ PC-3 CSCs showing the colocalization of sENO1 (green) and cortactin (red) at the ventral side of cell. Scale, 8 μm. Right upper, digital zoom-in image from serial Z sections (yellow rectangle) showing the spatial colocalization of sENO1 (green) and cortactin (red) at invadopodia. Scale, 5 μm. Right lower, the orthogonal view of the magnified areas (yellow squares at top) shown the distribution and localization of sENO1 and cortactin at the base of invadopodia. 3D rendered images of the invadopodia (arrows) were processed by using Imaris software. Scale, 1 μm.

In the original article, there was a mistake in **Figure 4** as published. We mislabeled the name of the cell line as “NCI-N87” at the lower panel of **Figure 4B**, which should be “AGS” as described in the main text. We mark the right name in the red rectangle in the corrected figure below.

The authors apologize for these errors and state that these do not change the scientific conclusions of the article in any way.

