# Supplementary material for: A Novel Invadopodia-Specific Marker for Invasive and Pro-Metastatic Cancer Stem Cells
Source: Front Oncol. 2021 May 31;11:638311. doi: 10.3389/fonc.2021.638311 (PMC8200852; doi:10.3389/fonc.2021.638311)
Supplement: Supplementary file 1 [file DataSheet_1.pdf]

Supplementary Material

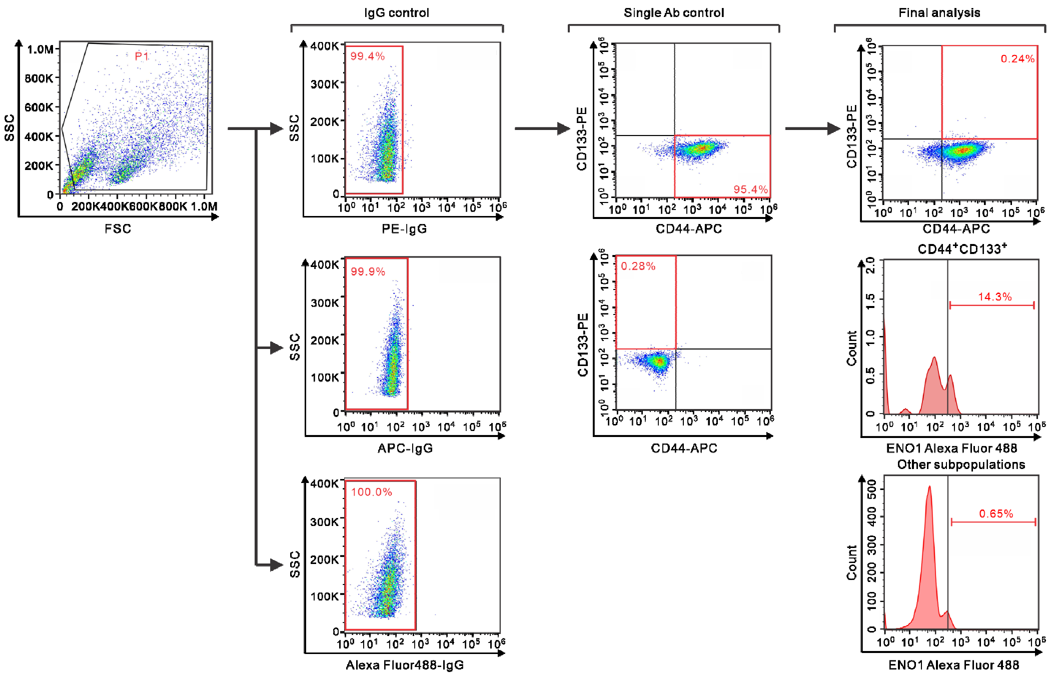

Supplementary Figure 1. Gating strategy and flow cytometric analysis of surface ENO1 (sENO1)$^+$ cells within CD44$^+$CD133$^+$ prostate adenocarcinoma cells. Shown are serial FACS plots demonstrating gating and analytical processes for patterns of ENO1, CD44, and CD133 staining of 22Rv-1 cells stained with anti-ENO1 or the isotype-matched control IgG. The cells were gated based on the cells stained with respective control IgG and then applied to the plots of cells stained with positive antibodies. Shown in the red boxes are the percentages of cells within the regions of interest.

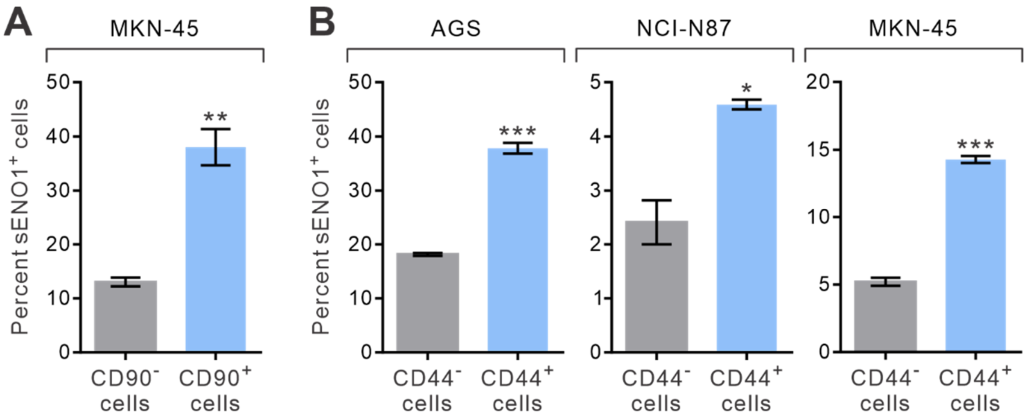

Supplementary Figure 2. Surface ENO1 (sENO1) is predominantly expressed by $CD90^+$ or $CD44^+$ cells in GAC cells. (**A**) The percentages of $sENO1^+$ cell subpopulation in $CD90^+$ or $CD90^-$ liver metastatic MKN-45 cells. (B) The percentages of $sENO1^+$ cell subpopulation in $CD44^+$ or $CD44^-$ AGS (primary GAC), NCI-N87 (liver metastatic GAC), or MKN-45 cells (liver metastatic GAC). Error bars represent mean ± SEM from three independent experiment (n = 3). Unpaired t-test was performed throughout where *$p < 0.05$; **$p < 0.01$; ***$p < 0.001$.

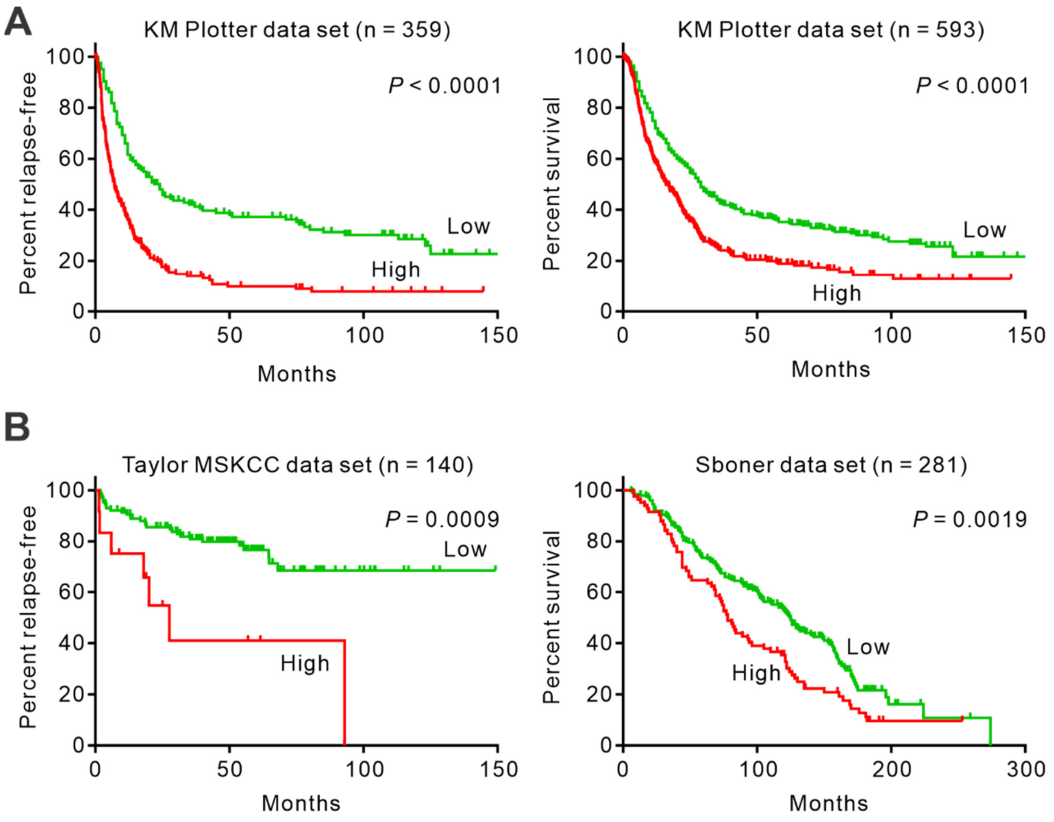

Supplementary Figure 3. ENO1 expression correlates with the poor prognosis in patients with GAC or PAC. (**A**) The relapse-free (left) and overall survival (right) of patients with GAC stratified based on the expression level of ENO1 as interrogated from KM Plotter (http://kmplot.com/analysis/). A log-rank test was performed. (**B**) The relapse-free (left) and overall survival (right) of patients with PAC stratified based on the expression level of ENO1 as interrogated from the indicated data sets. A log-rank test was performed.

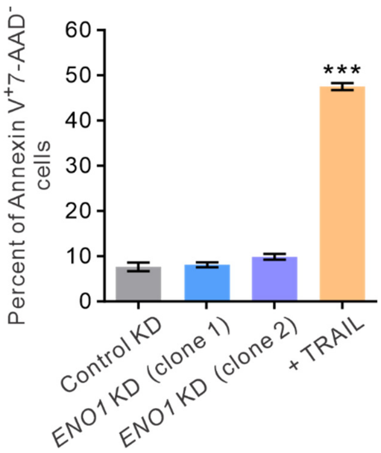

Supplementary Figure 4. The viability of prostate adenocarcinoma PC-3 cells with KD of *ENO1* expression. The cells were cultured in the presence of sodium pyruvate at the concentration of 100 μM for 3 days, after which their viability was determined by quantifying the Annexin V (ThermoFisher Scientific) and 7-aminoactinomycin D (7-AAD; BD Biosciences) staining patterns where Annexin V^{+}7-AAD^{-} cells are considered apoptotic cells. Cells treated with the apoptotic inducer (TNF-related apoptosis-inducing ligand; TRAIL) was included as a positive control. Unpaired t-test was performed throughout where ***$p < 0.001$ *versus* control KD.

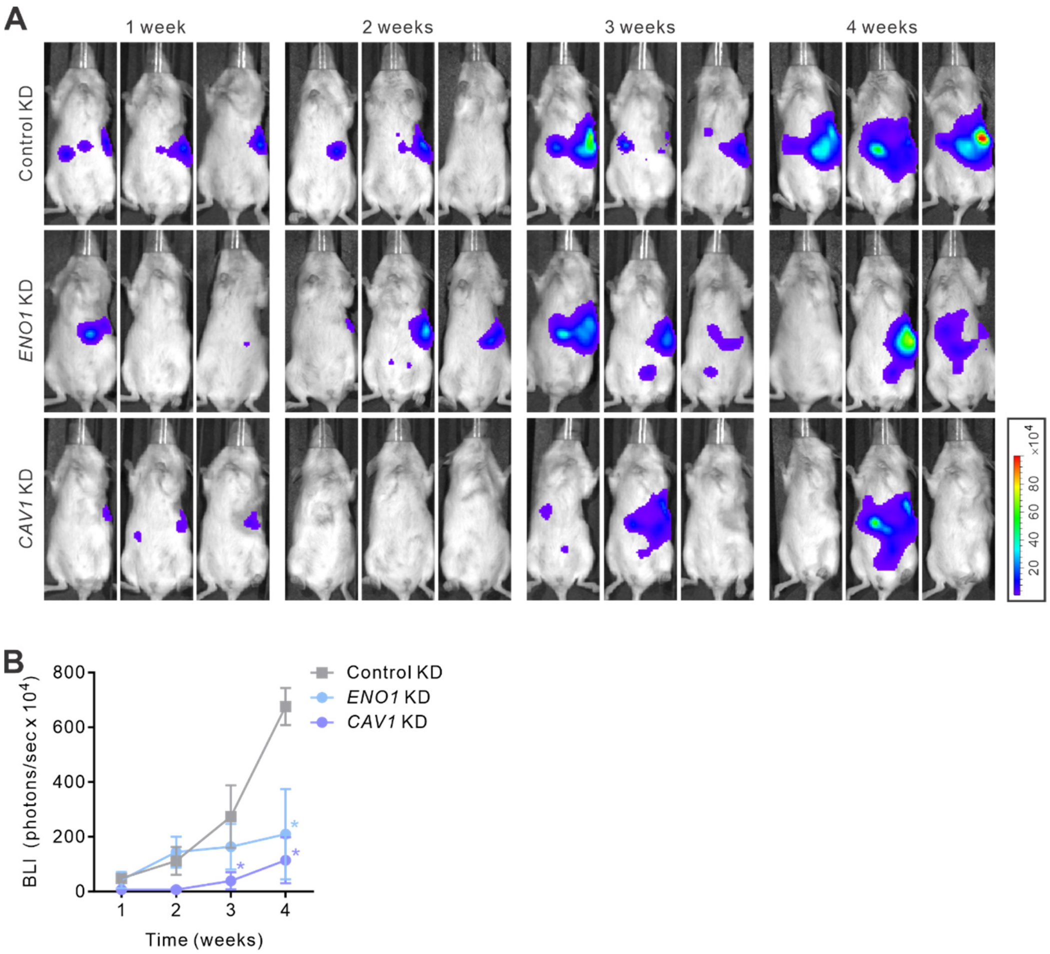

Supplementary Figure 5. ENO1 and CAV1 contribute to the pro-metastatic capability of GAC cells. (**A**) Representative bioluminescence images (BLI) of NOC/SCID mice receiving an intra-splenic injection of MKN-45 GAC cells with lentivirus shRNA-mediated knockdown (KD) of *ENO1* or *CAV1* expression or those with control KD at the indicated time following cell inoculation. (**B**) Tumor bulk quantified as BLI normalized photon counts as a function of time. Error bars represent mean ± SEM from one experiment (n = 8 mice per group). Unpaired t-test was performed throughout where *$p < 0.05$ *versus* control KD.
